# Stigmata of Degeneration and the Pregnesis of Epilepsy

**Published:** 1906-05-12

**Authors:** 


### STIGMATA OF DEGENERATION AND THE PROGNOSIS OF EPILEPSY.

The eighty-eighth volume of the "Medico-Chirurgical Transactions" contains a paper by Dr. W. Aldren Turner dealing with the above subject and based upon a study of 100 consecutive cases of confirmed epilepsy in the colony of epileptics, Chalfont St. Peter. The author defines stigmata of degeneration as "structural deviations from the normal, arising during the course of development, in those who are the subjects of a neuropathic heredity. Although primarily anatomical there have been described physiological and psychical stigmata." The author, however, is only concerned with the anatomical varieties.

The stigmata dealt with and regarded as structural evidences of neuropathic degeneration are these:—

1. Facial, including nasal, deformity and asymmetry.
2. Deformities of the hard palate.

3. Dental anomalies and displacements.
4. Deformities of the external ears.

1. The facial deformities observed included inequalities of the two sides of the face, in whole or in part, irregularities of the nose not due to trauma, prognathism, and feeble or arrested development of the lower jaw.

2. The configuration of the hard palate is considered according to Clouston's classification, under three heads:—(*a*) The normal palate, an arched structure with a low, regular, and wide dome. (*b*) The "neurotic" palate which is higher than the normal and somewhat narrower, but has a fair dome-shaped arch. This type of palate is commonly seen in persons of a nervous temperament, in neurasthenics and sufferers from depression, migraine, or dipsomania. (*c*) The "deformed" palate, which presents various abnormal shapes, is very high, narrow, keel-shaped, and somewhat irregular, and has a roof exhibiting a V-shaped appearance. Amongst the 100 cases these types were represented with the frequency shown by the following table:—

Normal, 54 per cent.
Neurotic, 33 per cent.
Deformed, 11 per cent.

Two cases presented the Torus palatinus, a mesial prominence along the palatal suture, occupying the whole or part of the palatal roof.

3. The dental anomalies consist in displacements of the teeth, most commonly of the lateral incisors.

4. Deformities of the ears are classified by the author as appears in the following table, which shows the relative frequency of the different departures from the normal in the series of cases under consideration:—

Normal ears, 65 per cent.
Abnormal shapes, 17 per cent.
Abnormal size, 11 per cent.
Asymmetry, 5 per cent.

The three chief varieties of stigmata were represented as follows in the series of 100 cases. Facial asymmetry, 42 per cent.; abnormal palates, 46 per cent.; abnormal ears, 33 per cent.

Such stigmata appear with greater frequency among male than among female epilepts, a fact which the author regards as related to the greater immunity from mental deterioration exhibited by female epileptics. Direct parental heredity of epilepsy or insanity is usually associated with more pronounced stigmata than is collateral heredity. When the cases are considered from the point of view of the age at which the disease first manifested itself, it is found that those affected during the first five years of life show a larger percentage of stigmata than any other class. This is in harmony with the observed fact that epilepsy commencing during the first hemi-decade is characterised by a tendency to profound mental impairment and liability to confirmation of the malady. But the absence of stigmata does not necessarily imply an early termination of the disease or a favourable outlook generally. There seems to be a definite relation between the presence of stigmata and the mental state. "Those epileptics who only showed the slighter degrees of mental impairment presented a nearly equal proportion with and without stigmata. On the other hand, of those in whom there was marked mental enfeeblement 53 per cent. exhibited them. Hence there would appear to be a close association between degrees of mental impairment and the presence of neuropathic stigmata, a fact which is proffered as an argument in favour of the view that the interparoxysmal mental condition in epilepsy is an integral part of the disease."

